# Relationship between nugent score and vaginal epithelial exfoliation

**DOI:** 10.1371/journal.pone.0177797

**Published:** 2017-05-31

**Authors:** Courtney P. Amegashie, Nicole M. Gilbert, Jeffrey F. Peipert, Jenifer E. Allsworth, Warren G. Lewis, Amanda L. Lewis

**Affiliations:** 1 Department of Molecular Microbiology, Washington University School of Medicine, St. Louis, MO, United States of America; 2 Center for Women’s Infectious Disease Research, Washington University School of Medicine, St. Louis, MO, United States of America; 3 Department of Obstetrics and Gynecology, Washington University School of Medicine, St. Louis, MO, United States of America; 4 Center for Reproductive Health Sciences, Washington University School of Medicine, St. Louis, MO, United States of America; 5 Department of Obstetrics and Gynecology, Indiana University School of Medicine, Indianapolis, IN, United States of America; 6 Department of Biomedical and Health Informatics, University of Missouri, Kansas City School of Medicine, Kansas City, MO, United States of America; 7 Department of Medicine, Washington University School of Medicine, St. Louis, MO, United States of America; Massachusetts General Hospital, UNITED STATES

## Abstract

**Objective:**

Clue cells characteristic of bacterial vaginosis (BV) are thought to arise due to exfoliation of the vaginal epithelium; however, there is little published data connecting total numbers of epithelial cells to markers of BV. The purpose of this study was to enumerate exfoliated epithelial cells (independent of clue cells) and examine the relationship to Nugent score.

**Study design:**

We conducted a cross-sectional sub-study of the Contraceptive CHOICE Project cohort. Vaginal swabs were used to create vaginal smears for Gram staining and these smears were later scored using the Nugent method, and then two blinded observers used microscopy to enumerate exfoliated epithelial cells. The degree of epithelial cell exfoliation was compared between women diagnosed as BV-negative (Nugent score 0–3), BV-intermediate (Nugent score 4–6), and BV-positive (Nugent score 7–10). BV specimens (Nugent 7–10) were randomly matched to specimens in the two other groups (Nugent low and Nugent-intermediate), in order to avoid comparing groups of women with potentially confounding baseline demographics.

**Results:**

Exfoliated epithelial cell counts were higher in the vaginal smears from BV-positive women compared with BV-negative women. Higher levels of epithelial exfoliation were also evident in BV-intermediate women compared to those with low Nugent scores. After adjustment for clustering introduced by matching, the incidence ratio of increased epithelial cell counts was 2.09 (95% CI 1.50–2.90) for the BV-intermediate women and 1.71 (95% CI 1.23–2.38) for the BV positive women.

**Conclusion:**

A vaginal epithelial exfoliation phenotype was measured in both Nugent-defined BV-positive and BV-intermediate women. Bacterial vaginosis and intermediate status (Nugent score >3) was associated with significantly more vaginal epithelial exfoliation compared to women with *Lactobacillus*-dominated microbiotas (Nugent 0–3).

## Introduction

The healthy human vagina is host to a *Lactobacillus*-dominated microbiota, which appears to have a critical role in thwarting colonization or infection by bacteria, viruses, and parasites that have been linked with negative health outcomes [1]. Approximately one in three women in the U.S. have bacterial vaginosis (BV) [2], a condition characterized by few lactobacilli, and an overgrowth of a diverse mixture of fastidious anaerobes. Many epidemiological studies have shown BV to be associated with increased risk of sexually transmitted infections (e.g., gonorrhea, chlamydia, HIV, trichomoniasis), and pelvic inflammatory disease [3–7]. In pregnancy, BV has been associated with infections of the placenta and amniotic fluid and with preterm birth [8–12]. However, despite the clinical implications of BV, little is known about its etiology.

BV is an imbalance of the vaginal microbiota and was defined in the 1980s as a diagnostic entity. In the clinical setting, BV may present asymptomatically, but it is often accompanied by increased pH, thin vaginal discharge, fishy odor, and the presence of “clue” cells, which are vaginal epithelial cells covered in bacteria [13]. Presently, the gold standard for laboratory-based BV diagnosis is the Nugent scoring method [14]. By this method, Gram stained smears of vaginal fluid are quantified for the presence of Gram positive ‘beneficial’ *Lactobacillus* morphotypes as compared to Gram negative organisms and Gram variable *Actinobacteria* such as *Gardnerella*, *Atopobium* and *Mobiluncus*. This approach results in an overall score in which 0–3 indicates a “normal” lactobacilli-dominated microbiota, 7–10 indicates BV, and the score of 4–6 has been referred to as an “intermediate microbiota” whose significance is incompletely characterized. Collectively, samples yielding a Nugent score greater than 3, i.e. both intermediate and BV together, have been referred to as “abnormal” vaginal flora or microbiota [7, 15, 16].

It has long been thought that clue cells, one of the clinical features of BV, are the result of an exfoliation of the vaginal epithelium in the presence of BV-associated bacteria [17, 18]. However, until recently there was little quantitative evidence to support this common assertion. Recently, we examined the quantity of epithelial cells present in human clinical vaginal smears and reported that specimens from women with Nugent-defined BV (score 7–10) exhibited significantly more exfoliated epithelial cells than women with a *Lactobacillus*-dominant microbiota (Nugent scores 0–3)[19].

This previous study had some potential limitations. First, there was no matching performed in the study. Given the higher risk of BV among African-American women, it is possible that race could be a confounder. The previous study also did not examine exfoliation across the entire range of Nugent scores (Nugent-defined intermediate scores of 4 to 6 were not studied). Interestingly, women with intermediate Nugent scores, like those with high Nugent scores, have been shown to have higher risks of sexually transmitted infections [7, 20–22], suggesting that shared features of the microbiology in women with Nugent scores greater than 3 may be responsible for increased susceptibility to secondary infection. Here, we sought to confirm the earlier finding of increased epithelial exfoliation among women with BV and to expand on the previous findings to determine if increased epithelial exfoliation is a shared feature of vaginal microbiomes with Nugent score greater than 3.

We used a matched cross-sectional design in which women defined as BV-positive (Nugent 7–10), BV-negative (Nugent 0–3), and BV intermediate (Nugent 4–6) were matched on the basis of age, race, and lifetime number of sexual partners. The aim of the present study was to quantify the extent of epithelial exfoliation in clinical specimens representing all three Nugent-defined categories.

## Materials and methods

### Study design

This is a cross-sectional study with matching that uses samples and questionnaire data from women enrolled in the Contraceptive CHOICE Project (CHOICE). CHOICE received approval from the IRB at Washington University School of Medicine. Written informed consent from all participants was obtained at enrollment, as well as permission to use vaginal samples for future studies. This sub-project received IRB approval (ID # 201108155) first in 2011; the most recent continuing review was in January 2016. Both the parent study (CHOICE) and this sub project were conducted according to the principles expressed in the Declaration of Helsinki.

From August 2007 to September 2011, a total of 9256 St. Louis-area women were screened and enrolled through CHOICE-affiliated clinical sites. CHOICE inclusion criteria were: reported sexual activity in the past six months or anticipated sexual activity with a male partner and the desire to prevent pregnancy by use of contraception. Exclusion criteria were ages outside the 14–45 year age range and history of tubal ligation or hysterectomy, as previously described [23].

Women who had a complete baseline survey and vaginal swab collected were eligible for this analysis. We excluded women who reported a race other than white or black. We note that few individuals enrolled in the CHOICE study self-identified in groups other than white” or black and that demographic characteristics of the CHOICE study were representative of the St. Louis region as a whole. Women with current *Chlamydia trachomatis*, *Neisseria gonorrhoeae*, *or Trichomonas vaginalis* infection were excluded to reduce the possibility of confounding variables that may also influence epithelial exfoliation. From the eligible individuals, 100 women with Nugent-defined BV (Nugent score 7–10) were chosen at random and were matched by age, race, and lifetime number of sexual partners to two controls, one in each of the Nugent categories “low” (0–3) and “intermediate” (4–6). In general, the Nugent scoring system combines bacterial morphotypes together with Gram staining properties to reflect the overall character of the vaginal microbiota [14]. Briefly, Nugent scoring occurs on a scale of 0 to 10 and has three components: 1) a score of 0–4 reflecting the presence of rod-shaped Gram positive lactobacilli where 0 indicates highest numbers, 2) a score of 0–4 reflecting the numbers of Gram negative and Gram variable bacteria where 4 indicates highest numbers, and 3) a score of 0–2 reflecting the presence of curved rods. Women were matched based on the risk factors of race and total number of sexual partners as well as by age, in order to avoid differences in vaginal physiology that may change over a woman’s reproductive years.

Matching was performed using a greedy matching algorithm; this approach selects a random match from the group of closest matches. Matches were exact match for race, had an age within 2 years, and a comparable number of lifetime number of sexual partners (exact matches to bins of 0–2, 3–7, 8–12, 13–17, 18–22, and 23+). After excluding matched sets in which one or more vaginal samples were not available for study, there were a total of 50 matched sets (150 women total). Our previous analysis of exfoliation suggested that we would be powered to observe differences in exfoliation with this number of matched participants.

Upon enrollment in CHOICE, women were instructed on a self-collection protocol and provided a double-headed rayon swab (Starplex Scientific Inc, Etobicoke, Ontario, Canada) for vaginal sample collection. One of these swabs was rolled on a glass slide which was Gram stained for Nugent scoring and epithelial exfoliation analysis. All swabs were rolled by the same technician to ensure consistency in application of biological material to the slides. As with any study performed on vaginal samples, different swabs will have somewhat different amounts of biological material. However, we note that all stained vaginal smears should contain some visible mucus, bacteria, and/or human cells. When this was not the case, we marked the samples as having too little biological material for proper analysis. Samples without an available Nugent score were excluded from our study.

### Epithelial exfoliation

Epithelial cell numbers were quantified from heat-fixed, Gram stained vaginal fluid slides as provided by CHOICE. Slides were visualized with a 10x objective on an Olympus BX61 microscope for a total magnification of 100x, and three representative images were captured from each specimen slide using StreamStart® software. Representative images were taken from locations on the slide that contained biological material. The images were not taken from locations on the slide near the start or end of the vaginal swab smears because these locations would have inconsistent and often large amounts of biological material. Exfoliated epithelial cells (all epithelial cells, not just clue cells) were then enumerated from the images by two independent observers who were blinded to Nugent score data. The three counts by two observers were summarized using a single harmonic mean, with counts of zero set to the limit of detection (one). Nugent scoring and epithelial enumeration were performed by different individuals. Epithelial cell enumerators never received training on Nugent scoring, and thus, were truly blinded to Nugent status.

In this study, we re-used Gram-stained slides previously employed for Nugent scoring and here analyzed the samples using a lower magnification. Exfoliated epithelial cells were enumerated using the same Gram stained slides used for prior Nugent scoring. As mentioned previously, the Nugent strategy is a validated and widely used method for bacterial morphotype scoring that provides a laboratory method for diagnosing BV. Thus, using the same slide for epithelial enumeration provides greater confidence that the numbers of epithelial cells are not solely a function of the amount of biological material present on a swab or deposited on a slide.

### Statistical analysis

**Fig 1:**
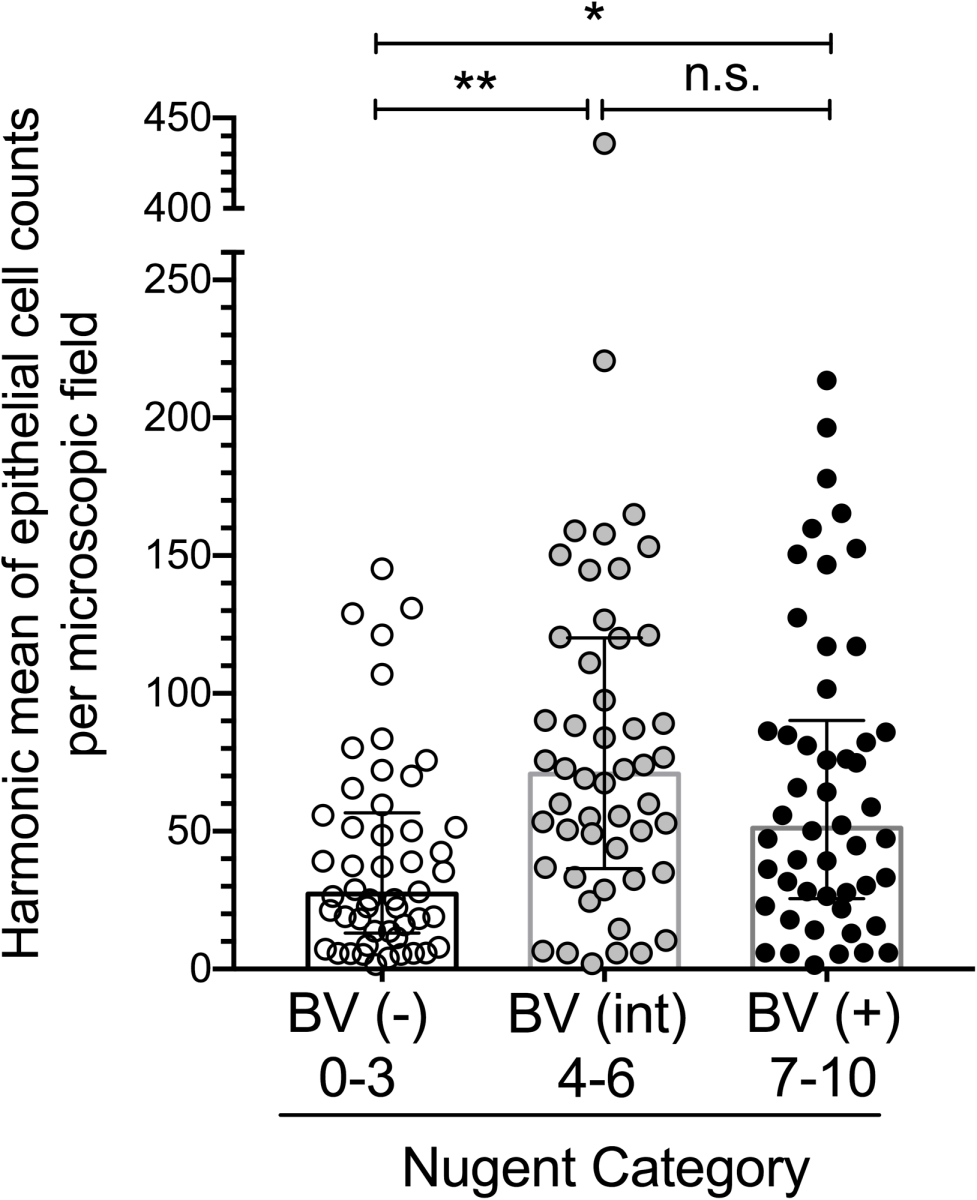
Relationship between Nugent score and epithelial exfoliation. Number of exfoliated epithelial cells by Nugent-defined BV group (negative, intermediate, and positive) is plotted as the harmonic mean of six counts from two observers. “Int.” = intermediate; “ns” = not significant. The central tendencies and error bars shown are medians and interquartile ranges respectively. The Friedman test (P < 0.01) was used to compare differences between groups, followed by Dunn’s multiple comparison post hoc test (** P < 0.01, * P < 0.05).

Categorical variables were compared using either chi-square statistics or Fisher’s exact tests, as appropriate. Potential confounders (listed in Table 1) were evaluated for association with outcome (epithelial exfoliation) and exposure (BV status/Nugent score). Criteria for confounding variables were: significance with both outcome and exposure at the alpha < 0.05 level for inclusion in adjusted regression models; and change in beta for the exposure variable of interest by 10% or more for inclusion in final models. No variables met these criteria, so unadjusted models are presented. Poisson regression models of epithelial counts were used to estimate incidence rate ratios (IRR) for BV status and Nugent score. Linear and Poisson regression models adjusted for clustering were used to factor-in the clustering introduced by matching BV positive specimens to negative and intermediate specimens using cluster-robust standard errors using the vce(cluster) option [24, 25]. These analyses were completed using Stata (version 13.1, Stata Corp, College Station, TX). GraphPad Prism 7.0 software was used to generate **Fig 1**. In addition to the analyses above, epithelial counts were compared between Nugent defined groups using the Friedman test (D’Agostino & Pearson normality test indicated that epithelial count data from all three groups were not normally distributed), followed by post hoc analysis using Dunn’s multiple comparisons test (analyses were performed in Prism).

**Table 1:** Characteristics of women from the Contraceptive CHOICE Project included in matched* analysis, N = 150.

	BV (-)	BV (int.)	BV (+)	P-value
N	50	50	50	
	N (%)	N (%)	N (%)	
Contraception prior to enrollment				0.79
No method	10 (20)	10 (20)	9 (18)	
Modern	18 (36)	13 (26)	13 (26)	
Condom	17 (34)	18 (38)	23 (46)	
Other	5 (10)	8 (16)	5 (10)	
Smoking status				0.48
Never	33 (66)	32 (64)	32 (64)	
Past	10 (20)	8 (16)	5 (10)	
Current	7 (14)	10 (20)	13 (26)	
Douched in past 30 days	2 (4)	4 (8)	6 (12)	0.34
Douched in past 180 days	5 (10)	10 (20)	14 (28)	0.07
Self-reported in past 7 days				
Discharge	7 (14)	9 (18)	8 (16)	0.86
Abnormal odor**	1 (2)	4 (8)	3(6)	0.53
Abnormal itching**	4 (8)	1 (2)	0	0.13

*Matched age (within two years), race, and number of sexual partners

**P-value using Fisher’s exact

## Results

### Participant characteristics

The demographic and reproductive characteristics of women in the study are shown in Table 1. The average age of participants was 25.2 years and 76% of participants self-identified as black. Nineteen percent of women reported 8–12 lifetime sexual partners, while 14% reported 13 or more partners. Thirty-five percent of women reported a history of smoking with 20% reporting current smoking at enrollment. Participants did not differ significantly by BV status in terms of current contraceptive method prior to enrollment, smoking history, douching in the prior 30 or 180 days or self-reported vaginal symptoms in the past 7 days (abnormal discharge, odor or itching, see S1 Table).

### Nugent scores greater than three are associated with higher epithelial cell numbers in vaginal fluid

Significantly higher numbers of exfoliated epithelial cells were measured in BV-positive women (Nugent 7–10) compared to their BV-negative (Nugent 0–3) counterparts (**Fig 1**). Nugent-defined intermediate status (scores 4–6) was also associated with significantly more epithelial exfoliation than matched BV-negative patients (**Fig 1**). Observed counts of exfoliated cells were modestly higher among the BV intermediate group compared with BV-positive participants, but there was no statistically significant difference between these groups. After adjustment for clustering introduced by matching, the incidence rate ratios for the BV intermediate and BV positive groups (compared to the BV negative group) were 2.09 (95% CI 1.50–2.90) and 1.71 (95% CI 1.23–2.38), respectively. In other words, women with Nugent scores of four or greater have ~1.7- to 2-fold more exfoliated epithelial cells than women with low Nugent scores. See S2 Table for the raw data underlying these findings. Demographic variables, types of contraception used, and behaviors such as smoking and douching were evaluated as potential confounders; however, none of these variables achieved significance for both outcome and exposure at the alpha < 0.05 level (see S1 Table).

## Discussion

Many studies of bacterial vaginosis describe the presence of clue cells as evidence of a vaginal epithelial exfoliation response. Here, we tested whether the *total* number of epithelial cells in vaginal specimens varied as a function of the vaginal microbiota (independent of the presence or numbers of clue cells). In this study, we show a correlation between total epithelial exfoliation (cell counts in vaginal specimens) and BV (women with Nugent scores of 7–10 had higher levels of epithelial cells compared to women with scores of 0–3). In fact, epithelial exfoliation was not specific to BV. Rather, women with Nugent scores greater than or equal to 4 had higher levels of epithelial cells in vaginal specimens compared to BV-negative women. We emphasize that our measures of exfoliation were *not* limited to epithelial “clue” cells; rather, all epithelial cells were counted to estimate the extent of exfoliation in relation to Nugent criteria.

The fact that BV-positive and BV-intermediate were phenotypically and statistically indistinguishable with respect to epithelial exfoliation is consistent with a role for Gram negative bacteria and *Actinobacteria* in BV pathophysiology. Typically, the difference between women who score as intermediate (4–6), versus those who have Nugent-defined BV (7–10) lies in the amount of lactobacilli present. Whereas intermediate women have some lactobacilli, women with BV have many fewer or absent lactobacilli. On the other hand, BV and intermediate women typically share the common feature of higher levels of bacteria that are phenotypically Gram negative or that vary morphologically from the distinct large rod-shaped lactobacilli. Thus, the presence of the exfoliation in Nugent intermediate women argues against the absence of lactobacilli as a driver of this phenotype. The finding that Nugent-defined BV and intermediate samples share an exfoliation response is consistent with a causal role for these organisms, whose overgrowth has been associated with BV.

Indeed, this interpretation parallels our previous findings in a mouse model, showing that *G*. *vaginalis* can elicit an exfoliation response of the vaginal epithelium [19]. In this previous model, we showed that infected animals exhibited a significantly higher number of epithelial cells in vaginal washes compared to mock infected animals. The animal model also exhibited several other clinical features of BV, including vaginal bacterial sialidase activity and vaginal mucus degradation.

Our analysis is based on stored specimens. We did not assess the association of exfoliation and Amsel’s criteria as these data were not readily available for analysis. Since clue cells have been defined according to their presence at the point of care in wet mount microscopy, which must be performed on fresh vaginal secretions, we were unable to perform a comparison of the amounts of clue cells in relation to the total number of exfoliated cells.

In the current study, epithelial counts and Nugent score were derived from the same Gram-stained slide examined at a different magnification. We acknowledge the possibility that BV could result in inherent differences in the type and/or quantity of biological material collected by vaginal swabbing, or that procedure of Gram-staining could influence epithelial cells differently because of inherent biological differences in samples from women with and without BV. However, as noted in the Materials and Methods, swabs take up a limited volume far below the total volume of vaginal fluid available. Studies by previous researchers estimate that women produce vaginal fluid at volumes between 4.5 to 7.9 mL per day [26], whereas swabs have a limited capacity at least an order of magnitude below this. Previous studies have shown a very good agreement between Nugent scores and Nugent-based BV diagnosis when comparing self-collected versus provider-collected samples, including studies involving pregnant [27] and nonpregnant [28, 29] women. Self-collected vaginal swabs also result in nearly identical 16S-rDNA-based bacterial community profiles as physician collected swabs [30]. Taken together, these findings argue that (1) uniform use of the same swabs (Starplex) across all women in the study should result in the collection of similar amounts of biological material, and (2) that the biological material sampled during self-collection of vaginal swabs is indistinguishable from clinician-collected material in terms of Nugent score and microbial community profiles. We also acknowledge that self-collection of vaginal swabs may limit the study by introducing more variation between samples, for example if some participants swab more rigorously than others. However, this is a concern that could also apply to contexts where multiple clinicians collect material from participants.

In the present study, possible confounding factors might include variables such as smoking status, which was previously shown to be associated with increased cervicovaginal epithelial exfoliation [31, 32]. Other studies have identified smoking as a risk factor for BV [33, 34]. Our study sample contained relatively few (20%) current smokers. Nonetheless, tobacco use and other potential confounders (listed in Table 1) were evaluated for association with outcome (epithelial exfoliation) and exposure (BV status/Nugent score). None of these variables achieved significance for both outcome and exposure at the alpha < 0.05 level (see S1 Table).

Another potential weakness of our study is a relatively low racial diversity of our participants, with 76% being black. As noted in the Materials and methods section, the demographic characteristics of the CHOICE study were representative of the St. Louis region (approximately half black and half white). Considering the fact that black women are differentially impacted by BV, they were over-represented in this study. Future studies would need to assess potential differences in exfoliation among different demographic groups from diverse geographic locations.

The relationship between exfoliation and intermediate Nugent score is a new finding with potential clinical implications. Recent studies using modern sequencing technology have measured the levels of different bacteria implicated in BV and compared these data across Nugent scores. These studies demonstrate that women with intermediate Nugent scores also tend to have high levels of BV bacteria measured by quantitative molecular methods [35, 36]. The finding of greater exfoliation in women with intermediate Nugent scores may explain previous findings that these women, like their BV-positive counterparts, are at increased risk of acquiring sexually transmitted infections (trichomoniasis, HIV, and gonorrhea) compared to women with Nugent scores 0–3[7, 20–22]. Our data suggest that epithelial exfoliation may be a host response to elevated numbers of BV bacteria, or the results of the action of bacterial virulence factors on epithelial cells, even if women do not meet the full Nugent criteria for BV (i.e. very low numbers of lactobacilli). Results of several previous studies suggest that certain detergents used in personal lubricants (e.g. nonoxynol-9) stimulate rapid epithelial exfoliation, a finding that may underlie the failure of such products to protect their users from HIV infection, despite their ability to disrupt viral membranes [37–40]. Further studies should explore whether epithelial exfoliation exposes niches in which sexually transmitted pathogens more easily gain or maintain residence in the reproductive tract.

## Supporting information

S1 Table Crude and adjusted associations with epithelial cell counts. (DOCX)

S2 Table A minimum raw dataset describing the results presented in the paper. This file contains randomized IDs, the matching sample IDs, Nugent status and the three epithelial counts from each observer.
(XLSX)
