# Supplementary material for: Relationship between nugent score and vaginal epithelial exfoliation
Source: PLoS One. 2017 May 31;12(5):e0177797. doi: 10.1371/journal.pone.0177797 (PMC5451030; doi:10.1371/journal.pone.0177797)
Supplement: S1 Table — (DOCX) [file pone.0177797.s001.docx]

Supplemental Table

Table A. Crude and adjusted associations with epithelial cell counts

| Variable | Crude iRR (95% CI) | Adjusted iRR (95% CI) |
| --- | --- | --- |
| Vaginal flora category |  |  |
| Normal flora | Referent | Referent |
| Intermediate flora | 2.09 (1.50, 2.90) | 2.00 (1.45, 2.75) |
| Bacterial vaginosis | 1.71 (1.23, 2.38) | 1.61 (1.13, 2.29) |
| Age (years) |  |  |
| 14-19 | Referent | Referent |
| 20-24 | 1.36 (0.86, 2.14) | 1.28 (0.86, 1.91) |
| 25-29 | 1.23 (0.84, 1.80) | 1.25 (0.89, 1.77) |
| 30 & older | 1.00 (0.67, 1.48) | 0.92 (0.64, 1.33) |
| Black race | 0.81 (0.58, 1.13) | 0.78 (0.55, 1.12) |
| Hispanic | 1.75 (1.23, 2.50) | 1.30 (0.84, 2.03) |
| Smoking status |  |  |
| Never | Referent | Referent |
| Past | 1.00 (0.70, 1.44) | 1.106 (0.72, 1.55) |
| Current | 1.18 (0.80, 1.74) | 1.17 (0.80, 1.72) |
| Current contraceptive |  |  |
| No method | Referent | Referent |
| Any modern | 0.99 (0.70, 1.39) | 1.09 (0.77, 1.55) |
| Condom | 1.11 (0.73, 1.69) | 1.15 (0.77, 1.70) |
| Other | 1.06 (0.63, 1.78) | 1.05 (0.63, 1.75) |
| History of recent abortion | 1.12 (0.68, 1.84) | 1.04 (0.60, 1.79) |
| Douching in past 30 days | 1.05 (0.60, 1.83) | 0.86 (0.45, 1.62) |
| Douching in past 180 days | 1.24 (0.92, 1.67) | 1.20 (0.84, 1.72) |

*Adjusted models include vaginal flora category and potential confounder
